# Newborn genetic screening is highly effective for high-risk infants: A single-centre study in China

**DOI:** 10.7189/jogh.13.04128

**Published:** 2023-10-13

**Authors:** Xin Wang, Yun Sun, Xian-Wei Guan, Yan-Yun Wang, Dong-Yang Hong, Zhi-Lei Zhang, Ya-Hong Li, Pei-Ying Yang, Tao Jiang, Zheng-Feng Xu

**Affiliations:** Genetic Medicine Center, Women’s Hospital of Nanjing Medical University, Nanjing Maternity and Child Health Care Hospital, Nanjing, Jiangsu Province of China, China

## Abstract

**Background:**

Newborn genetic screening (NBGS) is promising for early detection of genetic diseases in newborns. However, little is known about its clinical effectiveness in special groups like high-risk infants. To address this gap, we aimed to investigate the impact of NBGS on high-risk infants.

**Methods:**

We screened 10 334 healthy newborns from the general maternity unit and 886 high-risk infants from the neonatal ward using both traditional newborn screening (tNBS) and NBGS, and collected clinical data from electronic medical records.

**Results:**

We found that high-risk infants had a higher proportion of eutocia (*P* < 0.01) and prematurity (*P* < 0.01). For high-risk infants vs healthy newborns screened by tNBS, the primary screening positive rate was 3.84% vs 1.31%, the false positive rate (FPR) was 3.62% vs 1.18% (*P* < 0.001), and the positive predictive value (PPV) was 5.88% vs 8.27%. For NBGS vs tNBS in high-risk infants, the primary screening positive rate was 0.54% vs 3.68%, the FPR was 0.22% vs 3.47%, and the PPV was 60.00% vs 5.88%.

**Conclusions:**

We found that combined newborn screening can effectively reduce the FPR caused by the high-risk symptoms and improve the PPV in high-risk infants, sufficient for more accurately showing the true status of the disease.

Traditional newborn screening (tNBS) is an effective global public health program for detecting severe disorders early and can help prevent deaths and improve the quality of life of children with newborn diseases [1,2]. However, its effectiveness is limited to a small number of diseases and it demonstrates relatively high false-positive rate (FPR) and low positive predictive value (PPV) [3,4]. To address these shortcomings, researchers have proposed next-generation sequencing-based newborn genetic screening (NBGS) as a complement to tNBS [5-7]. However, current research on NBGS mainly focuses on ordinary newborns rather than its applicability in special populations, like high-risk infants.

High-risk infants admitted to the neonatal ward can be categorised by their conditions into two groups [8]. The first includes the critically ill, severely premature, and low birth weight infants, most of whom are admitted to the neonatal intensive care units (NICUs) [9-11]. The second group consists of infants with pathological conditions, like foetal intracranial haemorrhage, idiopathic perinatal infection, and neonatal hyperbilirubinemia, who are admitted to the general neonatal ward according to guidelines for the construction and management of neonatal wards in China [12]. Evidence has shown that rapid whole-genomic sequencing (WGS) technology is suitable for high-risk infants in the NICU, with positive results [13-16]. However, it is not easily scalable for high-risk infants due to the high cost, especially for non-critically ill high-risk infants admitted to the general neonatal ward after birth. Furthermore, due to the low number of diseases screened for in the current tNBS and the limitations of methods based on biochemical metabolite detection, it is still unclear whether the high-risk symptoms in these high-risk infants are related to pathogenic genes or solely caused by external factors.

The use of NBGS based on next-generation sequencing (NGS) for ordinary newborns has been shown to be both effective and valuable [6,7,17]. Therefore, we considered exploring the suitability and impact of NBGS for high-risk infants from general neonatal wards who cannot undergo rapid WGS, but may have high-risk symptoms related to pathogenic genes, to improve our understanding of these infants through the implementation process. To this end, we collected dried blood spot samples from high-risk infants in the general neonatal ward and performed both NBGS and tNBS, analysed and compared the results of the two sequencing procedures, and used our findings to determine the value of NBGS in this population while providing information for improving for screening, diagnosis, and treatment.

## METHODS

### Study population and data collection

We conducted the study in Nanjing Maternity and Child Health Hospital, Jiangsu Province, China, where 14 467 infants were born between 18 March 2022 and 21 November 2022. From this overall population, 11 200 infants participated in the combined screening approach, 10 334 of whom were newborns from the general maternity ward and 886 high-risk infants from the general neonatal ward. We collected clinical data, including high risk factors, gender, delivery mode, birth weight, and gestational age, from the electronic medical records.

The Ethics Committee of the Women’s Hospital of Nanjing Medical University (2021KY-071) reviewed and approved this study. We obtained written informed consents from all the participants.

### Heel blood collection

We collected 400 μL samples of heel blood to create two dried blood filter papers when the neonate was 48-72 hours old with full lactation. We used one dried blood filter paper to detect the biochemical indicators by tandem mass spectrometry (MS-MS) or time-resolved fluorescence and the other to detect the pathogenic genes by targeted capture-based NGS.

### tNBS based on the detection of biochemical indicators

Thirty-seven diseases can be investigated at our centre for 45 biochemical indicators so far (Table S1 in the Online Supplementary Document). The biochemical indicators that can be detected by MS-MS are C0, C2, C3, C3DC+C4OH, C4, C4DC+C5OH, C5, C5:1, C5DC+C6OH, C6, C6DC, C8, C8:1, C10, C10:1, C10:2, C12, C12:1, C14, C14:1, C14:2, C14OH, C16; C16:1, C16OH, C16:1OH, C18OH, C18:1OH, ALA, ARG, CIT, GLY, LEU+ILE, MET, ORN, PHE, PRO, SA, TYR and VAL. We used the NeoBaseTM Non-derivatized MS-MS kit (3040-0010Z, PerkinElmer, USA) according to the manufacturer’s guidelines to detect these biochemical indicators. First, we took a three millimetre diameter dry blood filter paper and placed it in a 96 well plate. We added a 100 μL of a mixture containing methanol and acylcarnitine isotopic internal standards to each well. The plate was sealed and incubated with shaking at 45°C (650-750 r/min) for 45 minutes. Afterward, we transferred 75 μL of the extracted solution to a V-shaped bottom detection plate, covered it with aluminium foil, and then analysed it using a MS-MS system consists of Waters 1525 Binary HPLC Pump, Xevo TQD Triple Quadrupole Mass Spectrometry and Waters 2777 Sample Manager (Waters, USA). 17α-OHP and TSH can be detected using the GSP® Neonatal 17α-OHP kit (B015-112Z, PerkinElmer, USA) and GSP® Neonatal hTSH kit (3301-0010Z, PerkinElmer, USA), respectively, which we applied following the manufacturer's guidelines and analysed using the GSP® 2021 Genetic Screening Processor (PerkinElmer, USA).

### NBGS based on targeted capture-based NGS

Ninety-four diseases and 164 genes could be detected by targeted capture-based NGS (Table S2 in the Online Supplementary Document), which includes 9889 SNVs and 801 CNVs. We extracted the genomic DNA (DNA) by a QIAamp DNA Blood Midi Kit (51185, Qiagen, Hilden, Germany), the concentration of which was measured using a NanoDrop spectrophotometer (Thermo Scientific, USA). We sheared genomic DNA into small fragments of 100-500 bp DNA using a Covaris LE220 ultrasonic instrument (Massachusetts, USA). Magnetic beads were used to isolate fragments of 150-200 bp. After purification and end repair, we performed A-tailing at the 3′ends and adapter ligation to complete the DNA library construction. Libraries were quantified using an Agilent Bioanalyzer 2100 (Agilent Technologies, Santa Clara, CA, USA). After A-tailing and ligation, we used a customised IDT xGen Lockdown probe to capture the target region sequences, after which we pooled and quantified the hybridisation library and included single-strand circularisation and rolling circle replication. We sequenced the circularised library using a high-throughput gene sequencer (MGISEQ-2000) after making dynabeads with a PE100 + 10 sequencing type.

### Bioinformatics analysis

We used the Illumina bcl2fastq to convert the raw high-throughput sequencing data from Bcl to Fastq format. Sequencing reads were aligned to the NCBI human reference genome (hg19/GRCh37) after filtering the low-quality reads. We used the GATK software to detect SNV/indels and an in-house developed algorithm from Huada Gene to detect DMD/thalassemia/SMN1 Ex7 del [18]. We obtained the frequency of variant sites in the normal population was obtained from dbSNP [19], the 1000 Genome Project [20], and the Genome Aggregation Database (gnomAD) [21].

We interpreted variants per the American College of Medical Genetics (ACMG) guidelines [22] and literature searches [23-25] based on the level of evidence supporting pathogenicity. We identified pathogenic genes and sites and correlated them with diseases through databases such as OMIM [26], ClinVar [27], and the Human Gene Variant Database [28], and used software such as VarCards [29], Mutation Taster [30], PolyPhen-2 [31], and PROVEAN [32] to predict the biological functions affected by the variants.

### Sanger sequencing

Positive variants identified by NGS were validated by Sanger sequencing. We extracted genomic DNA from the dried blood spots and the target region was amplified using specific primers, performing the reaction using Phanta Max Master Mix (Vazyme, China). Purified PCR products were sequenced and analysed by capillary electrophoresis using an ABI Prism 3500XL Genetic Analyzer (Thermo Fisher, Waltham, MA, USA).

### Statistical analysis

We analysed data in R, version 4.2.2 (R Core Team, Auckland, New Zealand). We presented data as means and standard deviations (SDs) and performed a Pearson χ^2^ or Fisher exact test for statistical comparisons. We calculated the Kappa index based on the difference between how much agreement was present (“observed” agreement) compared to how much agreement would be expected to be present by chance alone (“expected” agreement) [33].

## RESULTS

### The screening efficacy (NBGS vs tNBS)

**Figure 1:**
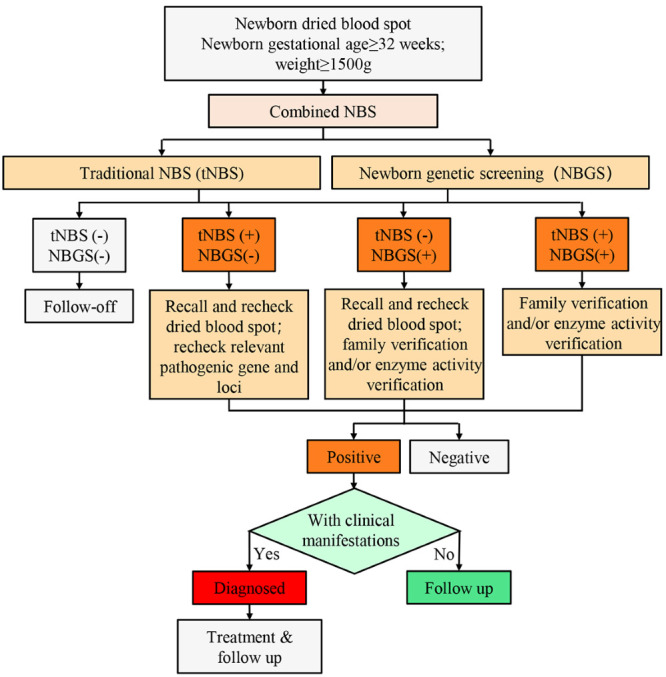
Newborn screening flowchart. The “-” symbol denotes negative and the “+” symbol positive values. tNBS – traditional newborn screening, NBGS – newborn genetic screening.

Overall, 94 genetic diseases could be detected by NBGS, significantly more than by tNBS (37 diseases) (**Figure 1**, Tables S1-S2 in the **Online Supplementary Document**). The difference of FPR is not significant between NBGS and tNBS (1.50% vs 1.37%), but the diagnosed ratio (0.51% vs 0.12%) and PPV (25.45% vs 7.78%) of NBGS were higher than tNBS (Table S3 in the **Online Supplementary Document**), Kappa-value of NBGS and tNBS suggested that the NBGS may improve the screening effectiveness compared to tNBS (0.37 vs 0.13).

### The impact of external delivery factors on high-risk symptoms of high-risk infants

**Figure 2:**
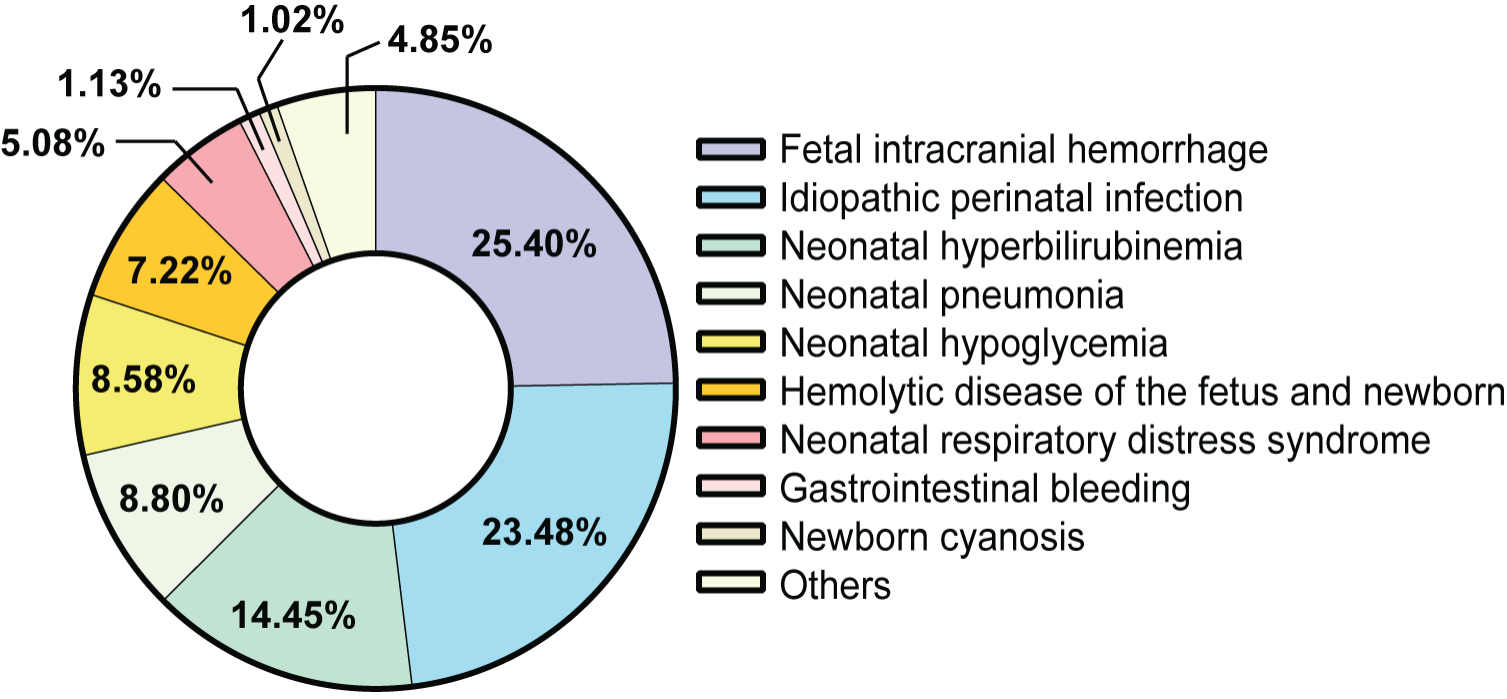
Proportion of each high-risk factor among high-risk infants.

A total of 10 334 healthy newborns and 886 high-risk infants participated in the combined screening approach, in which high-risk infants accounted for about 7.90%. The high-risk factors among the recruited high-risk infants include neonatal intracranial haemorrhage, idiopathic perinatal infection, neonatal hyperbilirubinemia, and others (**Table 1**). Among these factors, cerebral haemorrhage, and idiopathic perinatal infection were the most common, while other factors, like neonatal intestinal obstruction, pneumothorax, and respiratory distress syndrome were relatively rare (**Figure 2**). We found significant differences between high-risk infants and healthy newborns in the mode of delivery and gestational age (**Table 1** and Figure S1 in the **Online Supplementary Document**).

**Table 1:** Descriptive statistics of high-risk infants in the general neonatal ward and healthy newborn*

		Gender			Delivery mode			Weight				Gestational age		
**Classification**	**Total†**	**Male**	**Female**	***P-*value**	**Eutocia**	**Caesarean**	***P*-value**	**<2500 g**	**2500 ~ 4000 g**	**>4000 g**	***P-*value**	**<37 weeks**	**37 ~ 42 weeks**	***P-*value**
Healthy newborns	10334	5357 (51.84)	4977 (48.16)		5844 (56.55)	4490 (43.45)		15 (0.15)	9791 (94.75)	528 (5.11)		394 (3.81)	9940 (96.19)	
High-risk infants	886	502 (56.66)	384 (43.34)	<0.01	612 (69.07)	274 (30.93)	<0.001	2 (0.23)	830 (93.68)	54 (6.09)	0.372	51 (5.76)	835 (94.24)	<0.01
Fetal intracranial hemorrhage	225 (25.40)	139 (61.78)	86 (38.22)	<0.01	179 (79.56)	46 (20.44)	<0.001	0	212 (94.22)	13 (5.78)	0.743	20 (8.89)	205 (91.11)	<0.001
Idiopathic perinatal infection	208 (23.48)	114 (54.81)	94 (45.19)	0.396	157 (75.48)	51 (24.52)	<0.001	0	197 (94.71)	11 (5.29)	0.906	0	208 (100.00)	<0.001
Neonatal hyperbilirubinemia	128 (14.45)	64 (50.00)	63 (50.00)	0.746	93 (72.66)	35 (27.34)	<0.001	1 (0.78)	120 (93.75)	7 (5.47)	0.208	9 (7.03)	119 (92.97)	0.099
Neonatal pneumonia	78 (8.80)	44 (56.41)	34 (43.59)	0.421	66 (84.62)	12 (15.38)	<0.001	0	72 (92.31)	6 (7.69)	0.371	0	78 (100)	0.144
Neonatal hypoglycemia	76 (8.58)	41 (53.95)	35 (46.05)	0.714	8 (10.53)	68 (89.47)	<0.001	0	68 (89.47)	8 (10.52)	0.157	12 (15.79)	64 (84.21)	<0.001
Hemolytic disease of the fetus and neonates	64 (7.22)	33 (51.56)	31 (48.43)	0.965	48 (75.00)	16 (25.00)	<0.01	1 (1.56)	60 (93.75)	3 (4.69)	0.109	3 (4.69)	61 (95.31)	0.971
Neonatal respiratory distress syndrome	45 (5.08)	26 (57.78)	19 (42.22)	0.426	28 (62.22)	17 (37.78)	0.444	0	41 (91.11)	4 (8.89)	0.336	2 (4.44)	43 (95.56)	1.000
Gastrointestinal bleeding	10 (1.13)	8 (80.00)	2 (20.00)	0.143	4 (40.00)	6 (60.00)	0.462	0	10 (100)	0	1.000	1 (10.00)	9 (90.00)	0.323
Newborn cyanosis	9 (1.02)	5 (55.56)	4 (44.44)	1.000	6 (66.67)	3 (33.33)	0.783	0	8 (88.89)	1 (11.11)	0.385	1 (11.11)	8 (88.89)	0.296
Others	43 (4.85)	28 (65.12)	15 (34.88)	0.082	23 (53.49)	20 (46.51)	0.686	0	42 (97.67)	1 (2.33)	0.742	3 (6.98)	40 (93.02)	0.496

*Data presented as n (%) unless specified otherwise.

†The ratio of neonates in neonatal unit.

Among the high-risk infants, neonatal hypoglycaemia was more common in newborns delivered by caesarean section, while the proportion of eutocia was higher in newborns with neonatal intracranial haemorrhage, idiopathic perinatal infection, neonatal hyperbilirubinemia, neonatal pneumonia, and neonatal haemolytic disease. Moreover, the proportion of intracranial haemorrhage and neonatal hyperbilirubinemia in premature infants in the general neonatal ward was significantly higher than that in newborn with a normal gestational age. There were no significant differences in sex and birth weight between high-risk infants and healthy newborns (**Table 1** and Figure S1 in the **Online Supplementary Document**).

The carrier rate and quantity of pathogenic genes in the high-risk infants were not significantly different from those of healthy newborns, according to an analysis of the NBGS of high-risk infants. GJB2, MMAHCC, and DUOXA2 all had greater carrier rates in high-risk newborns than in healthy newborns and GJB2 had the highest carrier rate of any pathogenic gene; however, it has not been discovered that high-risk infants under each high-risk factor have a clear preference for carrying a particular gene (Figure S2, panels A-C in the **Online Supplementary Document**), suggesting that more high-risk symptoms of high-risk infants may be brought on by the mode of delivery or premature birth rather than a specific disease caused by the pathogenic gene.

### High-risk infants are more likely to cause false positives in tNBS due to the existence of high-risk factors

**Figure 3:**
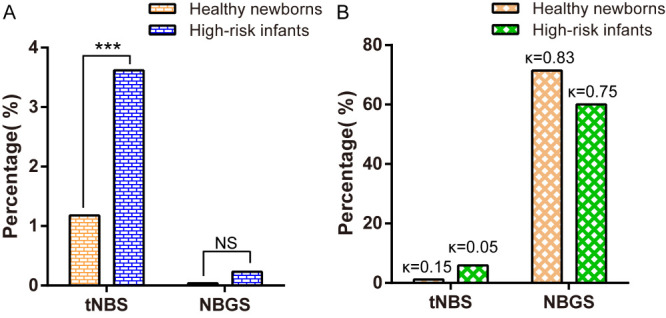
Comparison of tNBS and NBGS between healthy newborns and high-risk infants. **Panel A.** Comparison of FPR. **Panel B.** Comparison of PPV. Kappa values denote none to slight (0.01≤κ≤0.20), fair (0.21≤κ≤0.40), moderate (0.41≤κ≤0.60), substantial (0.61≤κ≤0.80), or almost perfect agreement (0.81≤κ≤1.00). ***P < 0.001. NS – not significant, κ – Kappa values.

Concerning genetic metabolic diseases covered by both NBGS and tNBS, the primary screening positive rate (3.84% vs 1.31%; *P* < 0.01) and FPR (3.62% vs 1.18%; *P* < 0.01) of tNBS for high-risk infants was significantly higher, while the PPV is lower than that of healthy newborns (5.88% vs 8.27%), consistent with the kappa index (0.05 vs 0.15). We further found that neonatal hypoglycaemia, gastrointestinal bleeding, and neonatal cyanosis had relatively high FPRs in high-risk infants. When compared with tNBS, NBGS demonstrated superior results with a kappa index of 0.75 vs 0.05. It significantly reduced the primary screening positive rate (0.56% vs 3.84%) and FPR (0.23% vs 3.62%) of high-risk infants, and significantly increased the PPV (60.00% vs 5.88%) (**Figure 3**). When compared with healthy newborns, the positive rate (0.56% vs 0.15%) and FPR (0.23% vs 0.04%) of NBGS for high-risk infants were higher than those of healthy newborns (**Table 2** and Table S4 in the **Online Supplementary Document**). The abnormal biochemical indicators that were positive in the initial screening of tNBS in the high-risk infants were mainly Cit, TSH, ORN and 17-α-OHP (Table S5 in the **Online Supplementary Document**).

**Table 2:** Results of traditional and genetic screening of the same disease among healthy newborns and high-risk infants*

		Traditional screening								Genetic screening							
	**Total cases**	**Number of positive cases**	**Ratio**	**Diagnosed cases**	**Ratio**	**FPR**	***P*-value**	**PPV**	**Kappa-value**	**Positive**	**Ratio**	**Diagnosed cases**	**Ratio**	**FPR**	***P-*value**	**PPV**	**Kappa value**
**All**	11 220	169	1.51%	13	0.12%	1.37%		7.78%	0.13	20	0.18%	13	0.12%	0.05%		68.42%	0.81
**Healthy newborns**	10 334	135	1.31%	11	0.11%	1.18%	<0.001	8.27%	0.15	15	0.15%	10	0.10%	0.04%	0.075	71.43%	0.83
**High-risk infants**	886	34	3.84%	2	0.23%	3.62%		5.88%	0.05	5	0.56%	3	0.34%	0.23%		60.00%	0.75

FPR – false positive rate, PPV – positive predictive value

*One high-risk infant has a false-negative result from the tNBS. Two healthy newborns who underwent traditional screening and one healthy newborn who underwent genetic screening failed to be recalled.

### The advantages of combined screening in high-risk infants

The NBGS of 886 high-risk infants found 15 positive cases in the initial screening, in which seven cases were finally diagnosed, three cases had no clinical manifestations, and five cases were excluded for different reasons, including family verification, and biochemical and enzyme activity tests (**Table 3**). There were two newborns that were confirmed positive by both tNBS and NBGS (No.9 and No.10). There was also one newborn whose tNBS of TSH was negative (No. 1, TSH = 2.2 mIU/L) but the NBGS was positive, while recalled for thyroid function testing was diagnosed with abnormal thyroid function. This indicated that the NBGS cannot only eliminate the false positives of tNBS caused by high-risk factors, but also effectively make up for missed screening outcomes caused by tNBS, suggesting that combined tNBS with NBGS can be sufficient for screening high-risk infants.

**Table 3:** Combined screening of positive newborn data in high-risk infants

High-risk factors	n	Gender	Traditional biochemical indicator	Gene	IP	Location	Genotype	Disease	Status
Fetal intracranial hemorrhage	1	Female	TSH = 2.2 mIU/L	PAX8	AD	EX4	c.280G>A	Congenital nongoiter hypothyroidism type 2	Diagnosed
	2	Male	-	TSC1	AD	EX17	c.2090_2091insTT	Tuberous sclerosis type 1	Unknown
Idiopathic perinatal infection	3	Male	-	GJB2	AR	EX2E, EX2E	c.235delC, c.187G>T	Autosomal recessive deafness type 1A	Diagnosed
	4	Male	-	GJB2	AR	EX2E	c.109G>A/, c.109G>A	Autosomal recessive deafness type 1A	Diagnosed
	5	Female	TSH = 1.34 mIU/L	DUOX2	AR	EX19, EX19	c.2524C>T, c.2654G>T	Thyroid secretion disorder type 6	Excluded
	6	Female	-	SCN1A	AD	EX25	c.4786C>T	Early onset infantile epileptic encephalopathy type 6/generalised epilepsy with febrile seizure type 2	Unknown
Neonatal hyperbilirubinaemia	7	Male	TSH = 7.86 mIU/L	DUOX2	AR	EX20, EX20	c.3329G>A, c.2654G>T	Thyroid secretion disorder type 6	Diagnosed
	8	Male	CIT = 4.91μmol/L, ORN = 35.83μmol/L	OTC	XL	EX10	c.1015G>C	Aminoglycoside-induced deafness	Diagnosed
Neonatal pneumonia	9	Male	-	GJB2	AR	EX2E, EX2E	c.109G>A, c.176_191delGCTGCAAGAACGTGTG	Autosomal recessive deafness type 1A	Diagnosed
	10	Male	-	FGFR1	AD	EX17	c.2267G>A	Kallmann syndrome type 2	Unknown
	11	Male	17α-OHP = 5.78μmol/L	CYP21A2	AR	EX7, EX7	c.923_924insT, c.844G>T	21-hydroxylase deficiency	Excluded
Neonatal hypoglycemia	12	Female		G6PD	XLD	EX12	c.1388G>A	Glucose-6-phosphate dehydrogenase deficiency	Excluded
Neonatal respiratory distress syndrome	13	Male	-	G6PD	XLD	EX12	c.1388G>A	Glucose-6-phosphate dehydrogenase deficiency	Diagnosed
	14	Female	-	ATP7B	AR	EX2, EX15	c.588C>A, c.3316G>A	Hepatolenticular degeneration	Excluded
Hemolytic disease of the fetus and newborn	15	Female	-	G6PD	XLD	EX12	c.1388G>A	Glucose-6-phosphate dehydrogenase deficiency	Excluded

TSH – thyroid stimulating hormone, CIT – citrulline, ORN – ornithine, 17α-OHP – 17α-hydroxyprogesterone, IP – inheritance pattern, AD – autosomal dominant inheritance, AR – autosomal recessive inheritance, XL – X-linked inheritance, XLD – X-linked dominant inheritance

## DISCUSSION

We aimed to explore the application of NBGS in general neonatal ward high-risk infants, a unique population with specific high-risk factors compared to ordinary newborns, yet with relatively mild conditions when compared to NICU high-risk infants. Previous studies have demonstrated the usefulness of NBGS in the general newborn population, as it allows the screening for several diseases [34,35]. While the use of rapid WGS screening for NICU high-risk infants has been reported to be more effective [13,16,36], we found that it is not applicable in the general neonatal ward. This can be attributed to two main reasons; first, most high-risk infants in the general neonatal ward are not in as critical a state as those in the NICU, and second, most families of newborns in the general neonatal ward usually refuse rapid WGS due to its high cost. NBGS based on target capture-based NGS is supposed to be more applicable in this context. During our study, we found that the general neonatal ward high-risk infants are more likely to show false positives with tNBS, likely due to greater numbers of high-risk factors compared with healthy newborns. NBGS based on genetics cannot only eliminate the tNBS false positives caused by high-risk factors, but can also effectively make up for missed screening outcomes caused by tNBS, suggesting that the combination of tNBS with NBGS can be sufficient for screening high-risk infants in the general neonatal ward, as it can indicate the disease state more accurately.

Evidence from our earlier survey studies shows that individuals of childbearing age are greatly interested in the development of NBGS, expecting that NBGS can screen as many genetic diseases as possible and speed up the process of diagnosis and treatment [37]. Many studies have shown that genetic screening based on genome sequencing technology has a positive effect on newborn screening, such as expanding the screening process and increasing the detection rate of different diseases [38-40]. However, as China is a developing, low-middle income country with a dense population, genomic screening with its relatively high cost and complex technique is not yet applicable. We found that NBGS based on a target capture-based NGS technology might be more suitable in China, which is consistent with our previous retrospective study that also preliminarily demonstrated the necessity of NBGS [6] and its potential in compensating the existing limitations of tNBS. This study on NBGS for a special newborn population admitted to the general neonatal ward improved our understanding of the method’s clinical role and significant, and provides a reference for the development and improvement of newborn screening in developing countries.

We did not find a clear link between high-risk factors of high-risk infants and related pathogenic genes in the general neonatal ward. For example, we did not discover pathogenic genes linked to hyperinsulinemia in newborns with hypoglycaemia. It seems that most of the abnormalities in tNBS of high-risk infants in the general neonatal ward are not caused by genetic diseases, but by external factors. Combined newborn screening can resolve these issues, and can help avoid over-medication and reduce the psychological burden on family members. We also found that the diagnosis rate of high-risk infants is indeed higher than that of healthy newborns, and that the use of combined newborn screening in high-risk infants has a lower FPR and a higher PPV than in healthy newborns (Table S3 in the **Online Supplementary Document**). By using combined newborn screening, the results of both biochemical and genetic tests can be comprehensively evaluated, making it easier to find infants with abnormal manifestations early and reducing the chance of missing a diagnosis. Also, combining NBGS with metabolomics and other multi-omics could lead to the discovery of new biochemical markers of diseases in the newborns in the future.

Although the development of NBGS has brought new improvement opportunities for tNBS, it has also exposed some limitations. For example, although the detection cycle of NBGS ( ~ 10 days) is much shorter than that of genetic diagnosis (1 ~ 2 months), it is still longer than tNBS's ( ~ 2 days). Moreover, NBGS based on targeted capture based NGS technology has significant cost advantages over WGS and whole-exome sequencing (WES), but these advantages diminish when compared to conventional biochemical screening techniques. Therefore, it is necessary to focus on how to optimise the screening, diagnosis, and treatment process, improve NBGS detection technology to reduce the costs and shorten the detection time, and further improve the efficiency of combined newborn screening to boost early diagnosis and enhance the treatment of newborn diseases.

In the neonatal unit, 28.33% (n/N = 742/2616) newborns were high-risk infants from the NICU and 71.67% (n/N = 1875/2616) were high-risk infants from general neonatal ward, a population that cannot be ignored when considering the proportion. However, we found that the screening rate of NBGS in the general neonatal ward was significantly lower (47.25%, n/N = 886/1875) than that in the general maternity ward (87.20%, n/N = 10334/11851), indicating that NBGS is not adequately applied in the general neonatal ward. Our research indicates that conducting NBGS in general neonatal ward is more beneficial than in general units. Therefore, the development of NBGS in general neonatal ward should be actively encouraged to reduce the FPR and the number of missed patients, as well as the need for re-examination and other supplementary diagnosis and treatment for newborns in general neonatal ward.

This study had several limitations. The single-centre nature of the study resulted in a small sample size, potentially leading to selection bias. Moreover, we have not investigated the attitudes of parents of high-risk infants towards NBGS and did not analyse the cost-effectiveness of this approach. Accordingly, the diseases included in the NBGS panel and the NBGS technology require improvement to allow better application in clinical practice. Multicentre studies with large sample sizes are needed for the development of combined newborn screening strategies for the high-risk infant admitted to the general neonatal ward in the future.

## CONCLUSIONS

Our study preliminarily indicates that newborn screening which combines NBGS with traditional biochemical screening can effectively reduce the FPR caused by the high-risk symptoms and enhance the PPV for high-risk infants, this providing a more accurate representation of their health status. This provides guidance for the implementation and optimisation of combined newborn screening programs which are currently being developed and will likely be complete in the future.

## Additional material


Online Supplementary Document
