# Supplementary material for: Newborn genetic screening is highly effective for high-risk infants: A single-centre study in China
Source: J Glob Health. 2023 Oct 13;13:04128. doi: 10.7189/jogh.13.04128 (PMC10569371; doi:10.7189/jogh.13.04128)
Supplement: Online Supplementary Document [file jogh-13-04128-s001.pdf]

Online Supplementary Document

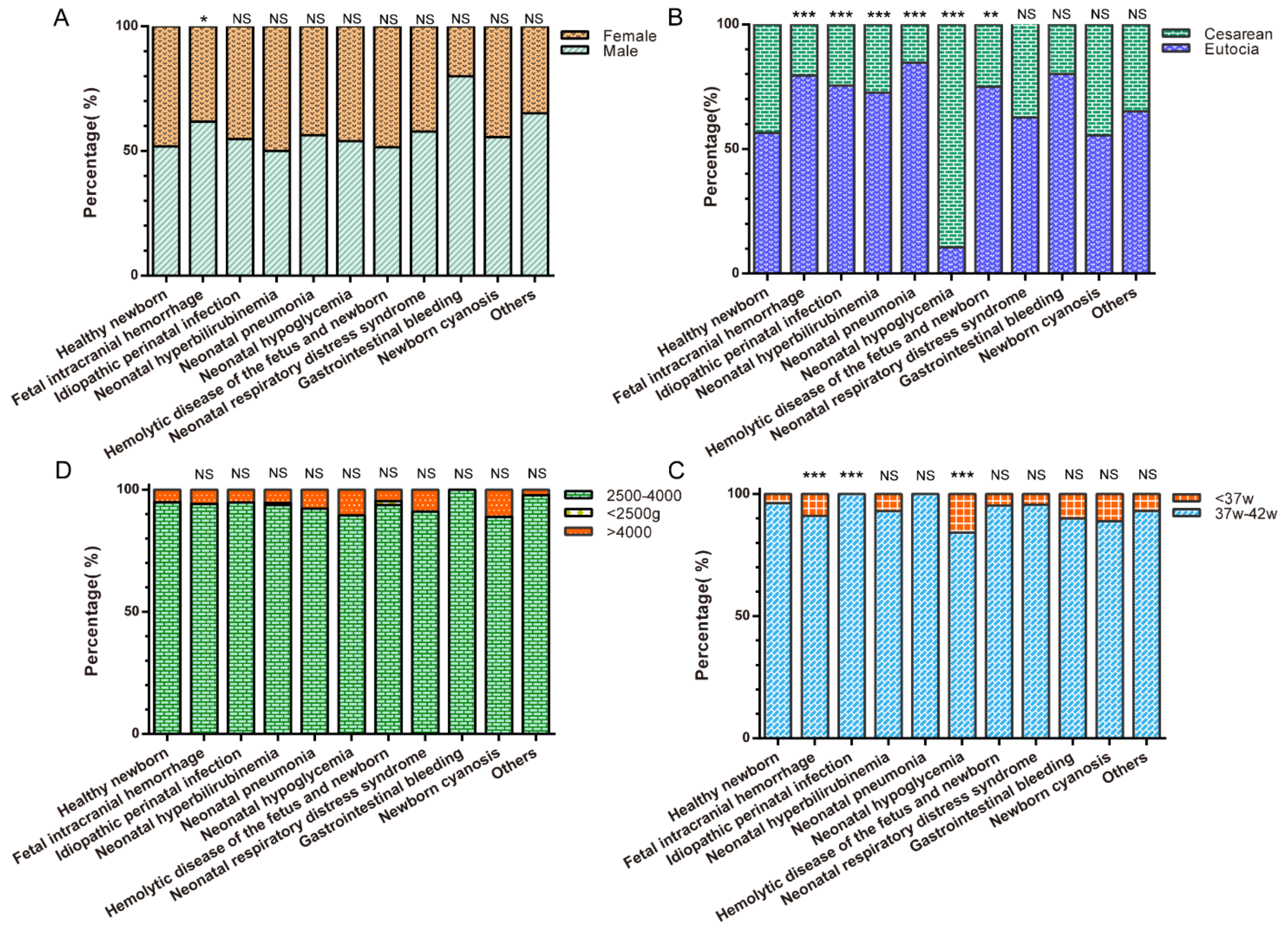

Figure S1. The effect of neonatal factors on high-risk infants. (A). The effect of neonatal gender on high-risk infants. (B). The effect of childbirth methods on high-risk infants. (C). The effect of neonatal weight on high-risk infants. (D). The effect of neonatal age on high-risk infants. *, P<0.05; **, P<0.01; ***, P<0.001; NS, not significant.

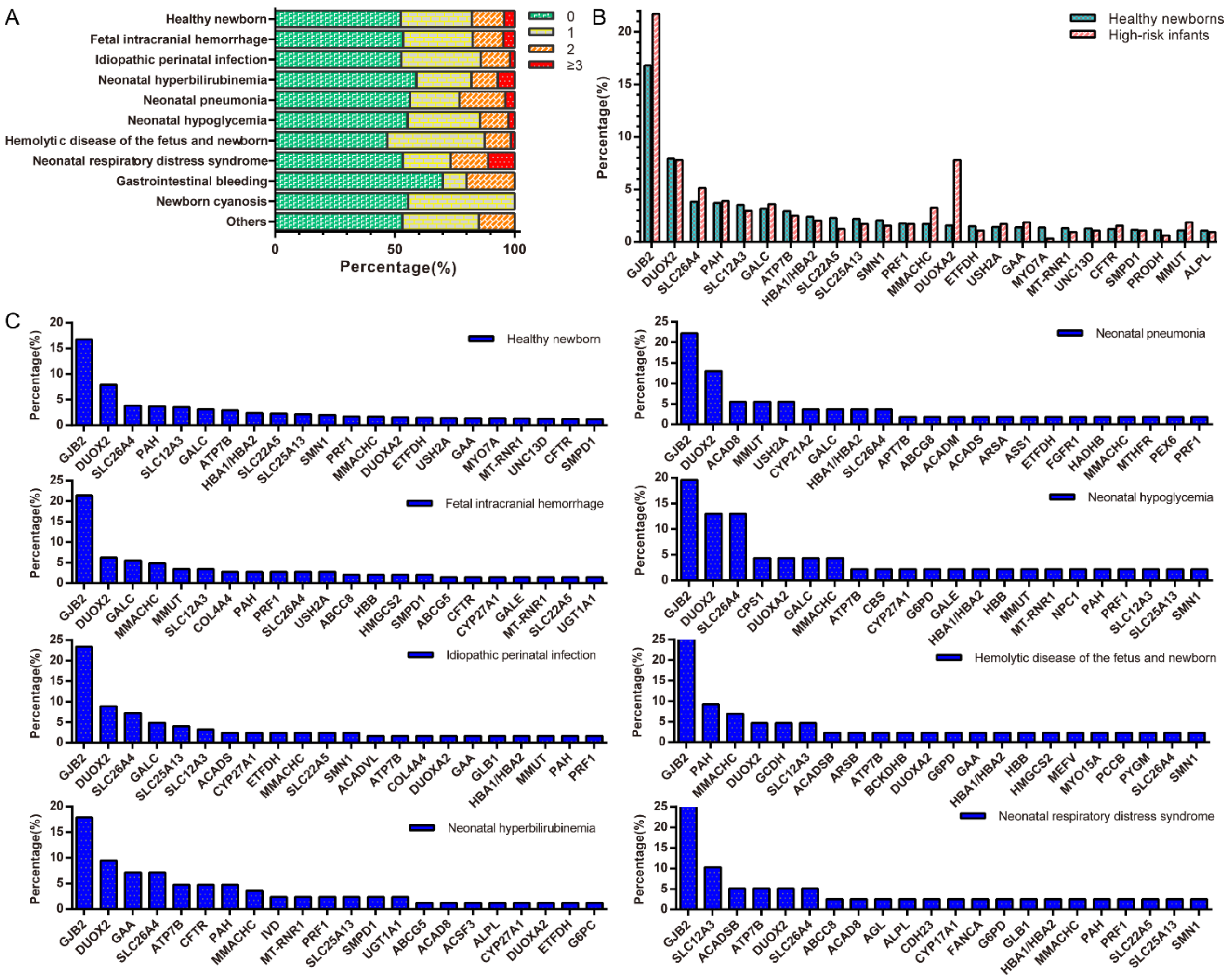

Figure S2. Pathogenic gene carrier status of high-risk infants. (A). The ratio of carried pathogenic gene numbers in high-risk infants. (B). The carrier rate of pathogenic gene in high-risk infants. (C). The carrier rate of pathogenic gene in different high-risk conditions.

Table S1. A list of the traditional biochemical screening diseases

Category	Disease	Detection method
Amino acid metabolism disorders	Hyperphenylalaninemia (HPA)<br>Maple syrupurine disease (MSUD)<br>Hyperprolinemia type 1<br>Homocysteinemia (HCY)<br>Tyrosinemia<br>Non-ketotic hyperglycinemia (NKH)<br>Hypermethioninemia	MS-MS
Urea cycle disorders	Citrin deficiency<br>Citrullinemia type I<br>Argininemia<br>Ornithine transcarbamylase deficiency (OTCD)<br>Carbamoyl phosphate synthetase I deficiency	MS-MS
Organic acid metabolism disorders	3-methylcrotonyl-coenzyme A carboxylase deficiency (MCCD)<br>3-hydroxy-3-methylglutaryl-coenzyme A lyase deficiency (HMGCL)<br>β-ketothiolase deficiency<br>Malonyl-coenzyme A decarboxylase deficiency<br>Propionic acidemia (PA)<br>Methylmalonic acidemia (MMA)<br>Biotinase deficiency (BTDD)<br>Glutaric Acidemia I (GA-1)<br>Isovaleric acidemia (IVA)<br>2-methylbutyrylglycinemia<br>3-methylpentolenoacidemia type I<br>Holocarboxylase synthase deficiency (HCS)<br>Isobutyryl-coenzyme A dehydrogenase deficiency	MS-MS
Fatty acid β oxidation disorders	Short-chain acyl-CoA dehydrogenase deficiency (SCADD)<br>Glutaric acidemia type II (GA-2)<br>Very long chain acyl-CoA dehydrogenase deficiency (VLCADD)<br>Primary carnitine deficiency (PCD)<br>Carnitine palmitoyltransferase deficiency (CPT)<br>Trifunctional protein deficiency (TFPD)<br>Long-chain 3-hydroxyacyl-CoA dehydrogenase deficiency (LCHAD)<br>Medium chain acyl-CoA dehydrogenase deficiency (MCAD)<br>2,4-Dienoyl-CoA reductase deficiency	MS-MS

	Carnitine-acylcarnitine translocase deficiency (CACT)	
Endocrine disease	Congenital adrenal hyperplasia (CAH)	Time-Resolved Fluorescence
	Congenital hypothyroidism (CH)	

Table S2. Genetic screening diseases and genes

Category	Disease	Gene	Genetic model
Amino acid metabolism disorders	Maple syrupurine disease (MSUD)	*BCKDHA*	AR
		BCKDHB	AR
		DBT	AR
	Tyrosinemia	*FAH*	AR
		TAT	AR
		HPD	AR
	Hyperphenylalaninemia (HPA)	*PAH*	AR
		PTS	AR
		QDPR	AR
	Homocysteinemia (HCY)	*CBS*	AR
		MTHFR	AR
	Non-ketotic hyperglycinemia (NKH)	*GLDC*	AR
		AMT	AR
	Hypermethioninemia	*MAT1A*	AR/AD
	Hyperprolinemia type 1	*PRODH*	AR
Urea cycle disorders	Argininemia	*ARG1*	AR
	Argininosuccinic aciduria (ASA)	*ASL*	AR
	Carbamoyl phosphate synthetase I deficiency	*CPS1*	AR
	Ornithine transcarbamylase deficiency (OTCD)	*OTC*	XLR
	Citrullinemia type I	*ASS1*	AR
	Citrin deficiency	*SLC25A13*	AR
	Hyperornithinemia-hyperammonemia-homocitrullinuria syndrome (HHHS)	*SLC25A15*	AR
Organic acid metabolism disorders	Methylmalonic acidemia (MMA)	*MMAA*	AR
		MMAB	AR
		MMACHC	AR
		MMUT	AR
	Propionic acidemia (PA)	*PCCA*	AR
		PCCB	AR
	Glutaric Acidemia I (GA-1)	*GCDH*	AR
	Holocarboxylase synthase deficiency (HCS)	*HLCS*	AR
	Biotinase deficiency (BTDD)	*BTD*	AR
	3-hydroxy-3-methylglutaryl-coenzyme A lyase deficiency (HMGCL)	*HMGCL*	AR

	Isovaleric acidemia (IVA)	*IVD*	AR
	3-methylcrotonyl-coenzyme A carboxylase deficiency (MCCD)	*MCCC1*	AR
		MCCC2	AR
	Malonyl-coenzyme A decarboxylase deficiency	*MLYCD*	AR
	3-methylpentolenoacidemia type I	*AUH*	AR
	2-methylbutyrylglycinemia	*ACADSB*	AR
	Isobutyryl-coenzyme A dehydrogenase deficiency	*ACAD8*	AR
	β-ketothiolase deficiency	*ACAT1*	AR
	Succinate hemialdehyde dehydrogenase deficiency (SSADHD)	*ALDH5A1*	AR
Fatty acid β oxidation disorders	Very long chain acyl-CoA dehydrogenase deficiency (VLCADD)	*ACADVL*	AR
	Long-chain 3-hydroxyacyl-CoA dehydrogenase deficiency (LCHAD)	*HADHA*	AR
	Trifunctional protein deficiency (TFPD)	*HADHA*	AR
		HADHB	AR
	Medium chain acyl-CoA dehydrogenase deficiency (MCAD)	*ACADM*	AR
	Short-chain acyl-CoA dehydrogenase deficiency (SCADD)	*ACADS*	AR
	Glutaric acidemia type II (GA-2)	*ETFDH*	AR
	Primary carnitine deficiency (PCD)	*SLC22A5*	AR
	Carnitine palmitoyltransferase deficiency (CPT)	*CPT1A*	AR
		CPT2	AR
	Carnitine-acylcarnitine translocase deficiency (CACT)	*SLC25A20*	AR
	2,4-Dienoyl-CoA reductase deficiency	*NADK2*	
Carbohydrate metabolism disorders	Glycogen storage disease (GSD)	*G6PC*	AR
		SLC37A4	AR
		GAA	AR
		AGL	AR
		GBE1	AR
		PYGM	AR
		PYGL	AR
		PHKA2	AR
		PHKB	AR
		PHKG2	AR
		PHKA1	AR
		PGM1	AR
	Galactosemia	*GALK1*	AR
		GALT	AR
		GALE	AR

Lipid metabolism disorders	Sitosterolemia	*ABCG8*	AR
		ABCG5	AR
	Familial hypercholesterolemia type 1 (FH)	*LDLR*	AD
Lysosomal storage diseases	Mucopolysaccharidosis (MPS)	*IDUA*	AR
		IDS	XLR
		SGSH	AR
		NAGLU	AR
		GALNS	AR
		GLB1	AR
		ARSB	AR
		GUSB	AR
	Krabbe disease	*GALC*	AR
	Fabry disease	*GLA*	XLD
	Niemann-Pick disease	*SMPD1*	AR
		NPC1	AR
		NPC2	AR
Mitochondrial disease	Mitochondrial encephalomyopathy with lactate acidosis and stroke-like episodes (MELAS)	*MT-TL1*	
		MT-ND5	
Hematological system diseases	α-thalassemia	*HBA1/HBA2*	AR
		HBA1	AR
		HBA2	AR
	β-thalassemia	*HBB*	AR
	Diamond-Blackfan anemia	*RPS19*	AD
		RPL11	AD
		RPS26	AD
	Fanconi anemia complementary group A	*FANCA*	AR
	Familial Hemophagocytic Lymphohistiocytosis (FHL)	*PRF1*	AR
		UNC13D	AR
	Glucose-6-phosphate dehydrogenase deficiency	*G6PD*	XLD
Skeletal system diseases	X-linked dominant hereditary hypophosphatemic rickets	*PHEX*	XLD
	Cartilage-Hair Hypoplasia	*RMRP*	AR
Neuromuscular disease	Spinal muscular atrophy	*SMN1*	AR
	Pyridoxine-dependent epilepsy (PDE)	*ALDH7A1*	AR
	Hereditary spastic paraplegia (HSP)	*REEP1*	AD
		ATL1	AD
		SPAST	AD
		SPG11	AD

	Congenital myotonia	*CLCN1*	AD
	Duchenne muscular dystrophy (DMD)	*DMD*	XLR
	Tyrosine hydroxylase deficiency (THD)	*TH*	AR
	Glucose transporter 1 deficiency syndrome (GLUT1-DS)	*SLC2A1*	AD
	Ohtahara syndrome	*SCN1A*	AD
		PCHD19	AD
Endocrine disease	Congenital hypothyroidism (CH)	*DUOXA2*	AR
		DUOX2	AR
		TSHR	AR
	Congenital adrenal hyperplasia (CAH)	*CYP11B1*	AR
		CYP17A1	AR
	Kallmann syndrome (KS)	*ANOS1*	XLR
		FGFR1	AD
		PROKR2	AR/AD
		CHD7	AD
	X-linked adrenal hypoplasia congenit (X-AHC)	*NR0B1*	XLR
	Combined pituitary hormone deficiency type 2	*PROP1*	AR
	Permanent neonatal diabetes mellitus	*KCNJ11*	AD
		ABCC8	AD
Intrahepatic cholestasis	Progressive Familial Intrahepatic Cholestasis	*ATP8B1*	AR
		ABCB11	AR
		ABCB4	AR
Hearing disorder	Hereditary non-syndromic deafness	*SLC26A4*	AR
		GJB2	AR
		GJB3	AD
		MYO15A	AR
		TMC1	AR
		TMPRSS3	AR
		OTOF	AR
		CDH23	AR
	Usher syndrome	*MYO7A*	AR
		PCDH15	AR
		USH2A	AR
	Mitochondrial non-syndromic sensorineural hearing loss (mNSSNHL)	*MT-RNR1*	
Immunodeficiency disease	Wiskott-Aldrich syndrome	*WAS*	XLR
	X-linked chronic granulomatous disease	*CYBB*	XLR
	X-linked angammaglobulinemia	*BTK*	XLR
	Severe combined immunodeficiency (SCID)	*IL2RG*	XLR
		RAG1	AR
	X-linked lymphoproliferative syndrom	*SH2D1A*	XLR

		XIAP	XLR
	Familial Mediterranean fever (FMF)	*MEFV*	AR
	X-linked hyperimmunoglobulin M syndrome (HIM)	*CD40LG*	XLD
	Severe congenial neutropenia (SCN)	*ELANE*	AD
Other metabolic related diseases	Bile Acid Synthesis Defect (BASD)	*HSD3B7*	AR
	Hepatolenticular degeneration (HLD)	*ATP7B*	AR
	Cerebrotendinous xanthomatosis (CTX)	*CYP27A1*	AR
	Menkes' disease (MD)	*ATP7A*	XLR
	Hypophosphatasia (HPP)	*ALPL*	AR/AD
Hereditary cancer related diseases	Retinoblastoma	*RB1*	AD
Other genetic diseases	Gitelman syndrome	*SLC12A3*	AR
	Leber hereditary optic neuropathy (LHON)	*MT-ND4*	
	X-linked Alport syndrome	*COL4A5*	XLD
	Alport syndrome	*COL4A3*	AD
		COL4A4	AR
	Tuberous sclerosis	*TSC1*	AD
		TSC2	AD
	Cystic fibrosis (CF)	*CFTR*	AR
	Co-Enzyme Q10 deficiency type 7	*COQ4*	AR

Abbreviations: AD, autosomal dominant inheritance; AR, autosomal recessive inheritance; XLD, x-linked dominant inheritance; XLR, x-linked recessive inheritance.

Table S3. Comparison of traditional newborn screening and genetic screening

	Total	Traditional screening							Genetic screening							
		Positive	Ratio	Diagnosed	Ratio	FPR[a]	PPV[b]	*Kappa-value*	Positive	Ratio	Diagnosed	Ratio	FPR	*P-value*	PPV	*Kappa-value*
All	11220	169	1.51%	13	0.12%	1.37%	7.78%	0.13	247	2.20%	57	0.51%	1.50%		25.45%	0.37
Healthy newborns	10334	135	1.31%	11	0.11%	1.18%	8.27%	0.15	232	2.25%	50	0.49%	1.55%	0.078	23.92%	0.35
High-risk infants	886	34	3.84%	2	0.23%	3.62%	5.88%	0.05	15	1.62%	7	0.76%	0.91%		46.67%	0.63

a, false positive rate; b, positive predictive value. One high-risk infant has a false negative result from the tNBS. Two healthy newborns who underwent traditional screening and one healthy newborn who underwent genetic screening failed to be recalled.

Table S4. Comparison of FPR and PPV with the different high-risk factors

	Total	Traditional screening					Genetic screening			
		Positive	Ratio	Diagnosed	FPR[a]	PPV[b]	Positive	Diagnosed	FPR	PPV
Fetal intracranial hemorrhage	225	3	1.33%	0	1.33%	0	2	1	0.46%	50.00%
Idiopathic perinatal infection	208	6	2.88%	0	2.88%	0	4	2	0.99%	50.00%
Neonatal hyperbilirubinemia	128	5	3.91%	2	2.34%	40.00%	2	2	0	100%
Neonatal pneumonia	78	2	2.56%	0	2.56%	0	3	1	2.60%	33.33%
Neonatal hypoglycemia	76	7	9.21%	0	9.21%	0	1	0	1.32%	0
Hemolytic disease of the fetus and newborn	64	1	1.56%	0	1.56%	0	1	0	1.56%	0
Neonatal respiratory distress syndrome	45	2	4.44%	0	4.44%	0	2	1	2.27%	50.00%
Gastrointestinal bleeding	10	1	10.00%	0	10.00%	0	0	0	0	0

Newborn cyanosis	9	1	11.11%	0	11.11%	0	0	0	0	0
Others	43	4	9.30%	0	13.95%	0	0	0	0	0

a, false positive rate; b, positive predictive value.

Table S5. The abnormal biochemical indicators in tNBS positive high-risk infants

High-risk factors	No.	Abnormal biochemical indicator (reference range)
Fetal intracranial hemorrhage	1	C0=6.57 (10-49.5umol/L)
	2	MET=38.21 (27.22μmol/L), TYR=21.08 (35-259μmol/L)
	3	CIT=5.98 (6-32μmol/L), ORN=30.48(52-369μmol/L), SA=0.41 (0.45-1.40μmol/L)
Idiopathic perinatal infection	4	CIT=4.32 (6-32μmol/L), TYR=27.65 (35-259μmol/L), GLY=225.73 (231-1055μmol/L), VAL=55.92 (56-233μmol/L)
	5	CIT=5.16 (6-32μmol/L), ORN=40.7 (52-369μmol/L), GLY=208.14 (231-1055μmol/L)
	6	TSH=9.44 (≤7.9mIU/L)
	7	17αOHP=13.4 (0-12nmol/L)
	8	TSH=9.34 (≤7.9mIU/L)
	9	C0=7.85 (10-49.5umol/L)
Neonatal hyperbilirubinemia	10	CIT=5.24 (6-32μmol/L), ORN=35.83(52-369μmol/L)
	11	TSH=8.44 (≤7.9mIU/L)
	12	C0=10.89 (10-49.5umol/L)
	13	ORN/CIT=3.824 (3.88-24.72)
	14	TSH=8.44 (≤7.9mIU/L)
Neonatal pneumonia	15	C0=10.89 (10-49.5umol/L)
	16	CIT=5.5 (6-32μmol/L), ORN=49.02 (52-369μmol/L)
Neonatal hypoglycemia	17	TSH=8.48 (≤7.9mIU/L)
	18	CIT=4.16 (6-32μmol/L)
	19	TSH=7.88 (≤7.9mIU/L)
	20	TSH=28.99 (≤7.9mIU/L)
	21	17αOHP=14.54 (0-12nmol/L)
	22	TSH=8.01 (≤7.9mIU/L)
	23	C2=40.59 (4.3-34.5), CIT=5.12 (6-32μmol/L),ORN=41.51 (52-369μmol/L)
Hemolytic disease of the fetus and newborn	24	C3=8.58 (0.38-3.6), C6DC=0.28 (0.05-0.27), TYR=26.89 (35-259μmol/L)
Neonatal respiratory distress syndrome	25	CIT=5.86 (6-32μmol/L)
	26	CIT=3.31 (6-32μmol/L), ORN=43.34 (52-369μmol/L), PRO=84.24 (86-330μmol/L)
Gastrointestinal bleeding	27	17αOHP=14.8 (0-12nmol/L), C0=8.31 (10-49.5umol/L)
Newborn cyanosis	28	TSH=8.64 (≤7.9mIU/L)
Others	29	17αOHP=24.78 (0-12nmol/L)
	30	C0=9.62 (10-49.5umol/L), CIT=5.87 (6-32μmol/L), ALA=120.9 (121-580μmol/L), ORN=27.84 (52-369μmol/L), GLY=207.49 (231-1055μmol/L), VAL=52.88 (56-233μmol/L), PRO=75.79 (86-330μmol/L)
	31	ORN/CIT=3.296 (3.88-24.72μmol/L), CIT=44.05 (6-32μmol/L), ALA=589.55 (121-580μmol/L)
	32	TSH=7.76 (≤7.9mIU/L)
